# Norwegian women’s experiences and opinions on contraceptive counselling: A systematic textcondensation study

**DOI:** 10.18332/ejm/132224

**Published:** 2021-02-03

**Authors:** Mirjam Lukasse, Marie Christine G. Baglo, Eldri Engdal, Ragnhild Lassemo, Kristin E. Forsberg

**Affiliations:** 1 Department of Nursing and Health Sciences, Faculty of Health and Social Sciences, University of South-Eastern Norway, Borre, Norway; 2 Maternity Unit, Tønsberg Hospital, Tønsberg, Norway; 3 Maternity Unit, Lillehammer Hospital, Innlandet Hospital Trust, Lillehammer, Norway; 4 Maternity Unit, Bærum Hospital, Vestre Viken Hospital Trust, Drammen, Norway; 5 Community Health Services, Horten, Norway

**Keywords:** contraceptive counselling, midwife, non-hormonal contraceptives, qualitative study

## Abstract

**INTRODUCTION:**

Contraception plays a pivotal role in most women’s lives, from teenage years to the menopause. Contraception and sexual wellbeing are closely related. Ideally, women should be able to access contraception and discuss issues concerning their sexual life during a contraceptive counselling session. Previously, only doctors conducted contraceptive consultations. Increasingly, other healthcare workers are providing contraceptive care. The aim of this study is to explore women’s experiences and opinions related to contraceptive counselling.

**METHODS:**

An electronic questionnaire was distributed in 2017–2018. The texts of 308 women’s written responses to open-ended questions were analyzed using systematic text-condensation.

**RESULTS:**

The analysis resulted in four themes: 1) Women-centered care, 2) Side-effects of hormonal contraceptives, 3) Non-hormonal methods and male involvement, and 4) Counsellors’ professional background. Women wished for a consultation that would lead to the best choice of contraception for them, taking into account their medical history, personal preference and living circumstances. Side-effects of hormonal products were under-communicated, as were non-hormonal methods. Respondents had contradicting opinions about midwives as contraceptive counsellors and were unfamiliar with them in this role.

**CONCLUSIONS:**

The quality of contraceptive counselling in Norway needs to be improved. Women require individualized follow-up, sufficient information and a choice of methods to find the most suitable alternative for them. A good relationship with a health provider they trust could improve contraceptive consultation. Midwives’ knowledge and competence in this area need to be made more widely known.

## INTRODUCTION

Generally, women are required to have a contraceptive consultation to get a prescription for regular hormonal contraceptives^1^. Contraceptive consultations can in addition offer women the opportunity to discuss other matters relating to sexual and reproductive health^2,3^. According to WHO the purpose of sexual healthcare should be the enhancement of life and personal relationships, and not merely counselling related to procreation or sexually transmitted diseases^3^. WHO further states that reproductive health implies that people are able to have a responsible, satisfying and safe sex life and that they have the capability to have children and the freedom to decide if, when and how often to do so^3^.

Despite the WHO definition of reproductive healthcare including the discussion of sexual wellbeing, the focus of most contraceptive consultations is providing appropriate contraceptives^4^. Which methods may be suitable depends on a woman’s medical history and risk factors, her preferences and her individual circumstances^5^. Thus, a contraceptive consultation needs to be long enough to take a proper history, provide information and discuss efficacy and side-effects^6^.

For health professionals and health authorities the efficacy (few women conceive) of Long-Acting Reversible Contraceptives (LARC) is one of the major reasons for promoting LARC as a first choice method^7^. A review of contraceptive use in the Nordic countries shows that the use of LARC has been increasing in these countries^8^. Unacceptable side-effects are the most common reason for discontinuing a specific contraceptive method^9,10^. The literature suggests that women do suffer considerable side effects, which are possibly under-communicated during the contraceptive consultation^1,11^. Non-hormonal contraceptive methods have a high Pearl Index (more women conceive) but less side-effects than hormonal contraceptives^12^.

Traditionally, medical doctors have been the primary providers of contraceptive care^1,13^. For most women the fertile period lasts about 30 years, starting at menarche and ending with the menopause. Thus, a considerable proportion of the population requires access to contraceptive care. In order to increase the prevention of unintended and unwanted pregnancies and the access to contraceptive care, a task-shifting and task-sharing process found place in a number of countries by which other health professionals were trained and authorized to provide sexual and reproductive care, including the prescription of hormonal contraceptives^13-16^. The role of midwives in this area has varied through time and from country to country^14,16-18^. However, providing counselling on family planning and methods of contraception are among the essential competencies midwives are expected to have by the International Council of Midwives (ICM)^19^.

Counselling skills are required to ensure women can make an informed choice about which contraceptive method to use^20^. Communication skills are pivotal to approach the sensitive topic of sexual health with women^21^. A genuine interest and acceptance that a woman is expert on her own life and body are a good foundation for a positive consultation experience^21^. Research shows that women find it hard to discuss intimate aspects of their life with health professionals and that some women may find it easier to discuss sexually related issues with a female health professional^1,21,22^. A good counsellor–patient relationship facilitates the decision-making process^10,11^.

When planning this study and publishing the quantitative results we discovered that there is limited research on women’s experience with contraceptive consultations in Norway^17^. We found only one recent qualitative study that included Somali women only^23^. The aim of our study was to explore women’s experiences and opinions related to contraceptive consultations.

## METHODS

### The Norwegian setting

Since 2002, Norwegian youth aged 15–20 years have had access to free consultations on sexual health and contraceptives through the public health youth clinic or school health services. Here, youth have access to free or subsidized contraceptives, including LARCs^24^. These services are primarily provided by public health nurses (PHNs) and some midwives. Until 2002, general medical doctors had monopoly on the prescription of contraceptives, with a fee for both the consultation and contraceptives^25^.

In 2016, midwives and PHNs with documented and registered education received the legal authority to subscribe contraceptive hormones to all women of fertile age^25^. Theoretically, it should have become easier for women to access contraceptive care. However, due to limited number of midwives and PHNs and the prioritization of other tasks such as antenatal and postnatal care and well-baby clinics, most public health centers still only offer this service for women aged <20 years.

Postpartum, on discharge or at the early home-visit, women may be advised by the midwife on contraceptives. Usually, women need to make an appointment, most often with their GP to access contraceptives.

### Method, design and sample

From 7 December 2017 to 28 February 2018, data were collected through an online questionnaire. The questionnaire was distributed with a link to Questback.com via Facebook pages such as SexandSociety.no, women’s groups, as well as political and student organizations. Inclusion criteria were women in fertile age from 15 years of age without any upper limit defined. To ensure anonymity, background information collected was limited to age, employment status, and size of municipality women lived in. The questionnaire consisted of two parts. The first part included quantitative questions regarding access and information given in contraceptive consultations and questions about sexual wellbeing. The quantitative results have been published previously^17^. The second part asked women to share ‘any other thoughts or issues they considered important regarding health personnel, contraceptive consultation, contraceptives and sexuality/sexual wellbeing’. The results of the quantitative data collected in the first part have been published^17^. The present study analyzes the qualitative data from the second part of the questionnaire.

A total of 1917 participants filled in the questionnaire (flowchart provided in Supplementary file). The total amount of text consisted of 42509 words. We disregarded comments that did not contribute to the purpose of this study, either because they were too short to allow interpretation or not relevant. This included statements like ‘great study’, ‘side-effects’ and ‘read the chapter on… in the book by….’. Having removed these, we were left with 35474 words from 308 women for the qualitative analyses. The length of these varied from one to 20 sentences. All sentences containing a meaning-bearing unit were kept.

### Qualitative analyses

The data were analyzed using systematic text-condensation, which is a descriptive and explorative method for thematic cross-case analyses of different types of data, also written texts^26^. The method has a pragmatic approach and consists of four steps. Although the process is described chronologically, in reality it is iterative^26^.

The second and third authors conducted the analyses under the supervision and with the contribution of the first author. Step one is called ‘total impression – from chaos to themes’^26^. All three authors read all the texts and discussed preliminary themes. The second step is called ‘identifying and sorting meaning units – from themes to codes’^26^. In this step, the text was read line-by-line to identify text fragments containing information about the research question, meaning units. Having identified the meaning units they were sorted into groups that fit together, code groups, themes and sub-themes. Step three is called ‘condensation – from code to meaning’^26^. In each theme, meaning units in all sub-themes were summarized and condensed. Step four is called ‘synthesizing – from condensation to description and concepts’^26^. The description is provided in the text of the results.

The Norwegian Centre for Research upon consultation evaluated the study as not requiring their approval. Our study followed the Helsinki Declaration of Ethical Principles^27^. Participants were informed of the purpose of the study and that responding to the questionnaire was considered consenting to the use of the data collected. Participants were informed and their anonymity was ensured, by not accessing their IP address at any stage, limiting the amount of background data collected and the way the data were collected. For example, age was collected in categories and not specific age. The data were stored at Questback.com initially and transferred encrypted to a password protected secure electronic storage. While analyzing the written texts, we ensured that when illustrating our results, we did not expose identity and maintained confidentiality.

## RESULTS

The majority of the women included in our study were aged 25–34 years and had used hormonal contraceptives during the past 6 months (Table 1). The analysis resulted in four themes: 1) Women-centered care, 2) Side-effects of hormonal contraceptives, 3) Non-hormonal methods and male involvement, and 4) Counsellors’ professional background. An overview of the themes and sub-themes is provided in Table 2.

**Table 1:** Background characteristics of the participants whose texts were included (N=308)

*Background characteristics*	*n*	*%*
**Age** (years)		
15–24	55	17.9
25–34	139	45.1
≥35	114	37.0
**Size of municipality by inhabitants**		
<30000	210	68.2
10000–30000	60	19.5
>30000	38	12.3
**Employment status**		
Employed	200	65.1
Student	74	24.1
Unemployed	12	3.8
Other	21	6.8
Missing	1	0.1
**Used hormonal contraceptives past 6 months**		
Yes	195	63.3

**Table 2:** An overview of the themes and sub-themes

*Themes*	*Sub-themes*
Women-centered care	How I would like to you to meet me. Take a history, you need to know about me. Provide information, do not tell me to Google it.
Side-effects of hormonal contraception	Emotional effects Physical effects Be honest about the side-effects.
Non-hormonal methods and male involvement	Inform us about other methods. Little acceptance for non-hormonal methods for contraception. Why are women responsible for contraception?
Counsellors’ professional background	Do midwives provide these services? Doctors know best. Practical challenges

### Women-centered care

Women wished for health professionals to meet them with interest, respect, and an open-mind. They wanted to be asked questions about their medical history and personal preferences. They felt it was important to develop a trusting relationship with the health professional. Sufficient time was necessary for a positive contraceptive consultation. Limited time hindered questions being asked, and good relations to be built. Women made it clear that talking about sexual health is challenging and difficult, not in the least to find the correct vocabulary. Some experienced the topic close to impossible, almost taboo, to discuss with health professionals.

‘As a woman who has given birth it seems that health professionals associated with this area of care are more concerned with the genitals' ability to give birth. Sexual health is a non-topic and a woman's knowledge of her own body is dismissed.’

There was no agreement among the participants on how women wished to be met by health professionals regarding sexual wellbeing. Some clearly did not want to be asked questions about sexual wellbeing. Their only reason for the consultation was to obtain contraception. Others considered the contraceptive consultation as a good opportunity to discuss other sexual wellbeing issues. However, the way the topic was approached was paramount. It should not be an interrogation or invasive. In addition, health professionals should be able to meet whatever arose in a conversation on sexual wellbeing. In particular, during the first consultation, the health professional should spend time to get to know the patient’s needs and medical background, including mental health.

‘I really wished that health staff to a larger degree had asked me how I was, and how my mental health was.’

Women want to find the contraceptive that suits them best, based on their medical history and living circumstances. There are many alternative options available and it is difficult to make a choice. Many had heard horror stories about some products that made them reluctant to try these products. The information provided at contraceptive consultations was insufficient to make an informed choice. Women had to search for information themselves and were even told to Google for information.

‘I have been surprised a long time at how poorly health professionals generally remember to give information, and ask the patient questions. First time I was to try oral contraceptives I was just given a type.’

### Side-effects of hormonal contraceptives

Hormonal contraceptives had unexpected side-effects. In particular, emotional side-effects were a surprise. Participants emphasized that there is little focus on the sideeffects during the contraceptive consultation. One woman became suicidal and started to self-harm shortly after she had a birth-control implant placed. Her mental health problems stopped almost completely after the contraceptive was removed. The connection between feeling low and using contraceptives was mentioned and exemplified by many of the participants. One woman described how hormonal contraception made her emotionally numb and depressed. This had a negative effect on her self-image, relationship with her partner and interest in sex.

‘I am not against adding hormones to my body, but paradoxically the contraceptive that should give med a sex-life without worries, has made a sex-life completely unthinkable.’

Physical side-effects were frequently mentioned. Some mentioned increased dysmenorrhea, others gaining weight. Irregular bleeding was a common problem. One woman experienced daily bleeding for seven months after the insertion of a birth control implant. When she changed to an Intra Uterine Device (IUD) these complications disappeared. Instead, she started having abdominal pain. The risk of deep vein thrombosis (DVT) appeared to be well known and many women noted fearing this particular complication. One woman experienced being prescribed contraceptives without her doctor taking a medical history to assess her risks, even though she had specifically inquired about serious side-effects. Later she developed DVT.

‘Think that soon I may have a baby as better alternatives are lacking. It seems that the only important thing is to prevent pregnancy, at the expense of side-effects and good health.’

A few women described having no side-effects. Many participants mentioned that some health professionals attempted to explain away side-effects and that their symptoms were not taken seriously. Follow-up after having started contraceptives was minimal.

### Non-hormonal methods and male involvement

Our findings show that women were requesting more information about non-hormonal contraceptive methods than provided. They experienced little acceptance for using or considering non-hormonal contraceptive methods, often called ‘alternative methods’. Some participants wanted information about safe periods, observing one’s own cycle, fertility apps, and copper IUD. One woman had a positive experience of using a Copper IUD and felt she had to defend this choice to her general practitioner (GP), given that she had no children and wanted this method. Many women felt that there was no room for discussing non-hormonal contraceptive methods with busy health professionals.

‘Good if you could stop pretending that hormonal contraceptives are the only solution and allow for one to actually say no on a medical basis and not just because one is deluded.’

Many participants mentioned that it was unfair that women have to bear the burden of contraception, often alone. Hormonal contraceptives for men were wanted. Participants writing about this also wrote that if men had to take hormonal contraceptives, they would not have accepted the kind of side-effects women endured. The question was posed on why there is little research on hormonal contraceptives for men. Sterilization could be an alternative way for men to take responsibility. The fact that this procedure is reversible and less invasive for men than women was described as an important advantage.

‘I just get so fed-up about us taking it for granted that it is a woman's job to use birth control. We have one good birth control method called a condom, and now we have to get men to take the burden. It sucks with rubber for you, but it sucks even more that I should disrupt my entire hormonal system so that you will have optimal sexual pleasure.’

### Counsellors’ professional background

The fact that midwives in Norway can prescribe hormonal contraceptives and are not just experts on birth but also on birth control was unknown to many of the participants, except for those from Sweden where midwives have had this role for several decades. While many participants were positive to consulting a midwife, some mentioned that for them it would be more logical to consult their GP who knew their medical history and has extensive knowledge of diseases. Others again experienced having to make an appointment for a contraceptive consultation with a male GP as a major barrier. Those participants unfamiliar with the option of attending a contraceptive consultation provided by a midwife were of the opinion that it should be made known more clearly that midwives do more than antenatal care. Most participants were positive about the midwives’ abilities to provide contraceptive care.

Several participants mentioned that it is easier to get an appointment with one’s GP than with a midwife. In addition, the location of the midwife’s office at the health center is regarded as an unnatural place to visit for birth control. A waiting room full of pregnant women does not make comfortable someone wanting birth control. In some areas only GP appointments are available and too few midwives are employed to even cover community antenatal care.

‘Completely new for me that midwives can place for example an IUD. Could be just as good even better alternative to a GP.’

## DISCUSSION

Our study showed that during a contraceptive consultation, women want to be asked relevant questions and given adequate information, including non-hormonal contraceptive methods. In particular, women wanted health professionals to be honest about side-effects of hormonal contraception. The midwife’s role in contraceptive counselling was not well-known.

Our study suggests that there is room for improvement in the quality of contraceptive consultations in Norway. The theoretical framework by Bruce and Jain^28^ suggests six aspects of contraceptive care to evaluate their quality^28^. We will discuss the findings from our study in relation to these aspects.

The first aspect is technical competence in the form of taking a history and assessing women’s needs, which are essential for finding the appropriate contraceptive method^28^. Women did not perceive that enough questions were asked about their medical background and personal needs. In our study, one reason general practitioners (GPs) did not ask as many questions as women thought necessary could be that they knew the women so well that no specific history taking was necessary. In Norway, a person is assigned to one GP for their lifetime. Another reason for limited assessment of women’s needs and history taking may be that the request for contraception comes at the end of a consultation that was not dedicated to contraception^29,30^, leaving very little time to deal with the contraception request. A dedicated consultation for contraception with sufficient time to exchange information seems a prerequisite for a high quality consultation^6^.

The second aspect to assess is the provision of information, including side-effects^28^. The women in our study expressed the wish for more information, in particular about side-effects. This is in agreement with other studies reporting that side-effects were under-communicated^1,11^. As side-effects are the primary reason for discontinuing contraception it seems of vital importance to prepare women^9,31,32^. Of all the different types of side-effects mentioned, the ones on mental health were most prominent and unexpected, according to our quantitative findings^17^. Several studies have reported the association of hormonal contraceptives and depression, use of anti-depressants, even self-harm, suicide ideation, and suicide^33,34^. However, there is no clear consensus if the effects are due to the hormonal contraceptives or due to the younger age of most users in which these mental symptoms are common^31,34^. Still our findings suggest that mental risk factors are not explored sufficiently.

As to the aspect of choice of methods presented, as reported previously in our study, women were given little information, except on non-hormonal methods^35-37^. While shared decision making and informed choice is promoted and encouraged in healthcare globally, it appears not so easy to accomplish in practice^38^. The health professionals focus, partly stimulated by national policy, on methods with a low Pearl Index seems to be in conflict with some women’s interest in non-modern, natural methods with a higher risk of pregnancy^36,38^. In addition, a GP may wonder why a woman consults them about contraception, if they do not need a medical prescription.

In the sub-theme ‘how I would like you to meet me’ women expressed the aspect of the consultation described under interpersonal relationships in the Bruce and Jain framework^28^. Several researchers have emphasized that health professionals need to have a genuine interest in the individual to provide tailored care^21,38^. Negative experiences with contraceptive counselling may affect contraception utilization^39^. Positive experiences with the contraceptive consultation and building a relationship of trust may provide the opportunity for a dialogue on sexuality and sexual abuse and other lifestyle issues^40,41^. High quality interactions appear to encourage contraceptive continuation^10,42^.

The aspect of continuity described by Bruce and Jain^28^ was not prominent in our findings. In Norway, women have to take the initiative to make a new appointment if they experience side-effects or want to change method. There is no systematic follow-up and women did not mention the need for it. For a continuation of the same prescription, they can just phone the practice and receive a new prescription without having to talk to the GP. The final aspect is the spectrum of services, covering also acceptability and appropriateness of counsellor and location^28^. The qualitative finding that women were unfamiliar with midwives as contraceptive counsellors is in line with our cross-sectional study^17^. The preference for a medical doctor, which some women expressed, may be due to their lack of experience with midwives in this capacity. In Norway, the extension of the midwife’s role in the area of contraceptives started in 2002, and became only extended to include all women by 2016^24,25^. So far, public services have not been extended sufficiently to allow all women to consult a midwife for contraceptive counselling. This is in contrast to neighboring Sweden where the majority of contraceptive counselling to healthy women is by midwives^4^. For many young women, consulting a midwife for counselling on contraceptives would allow them to have continuity of sexual healthcare throughout their reproductive life and assist them in planning their reproductive life^43^. Research suggests that adequately educated midwives/nurse-midwives can meet 90% or more of the need of sexual, reproductive, maternal, newborn and adolescent health, if they are part of a wider team operating within an enabled environment^16^.

### Strengths and limitations

We chose Malterud^26^ systematic text-condensation as it describes a pragmatic method suited for beginners^26^. Having several people involved in the analyses reduced the influence of an individual’s preconceived ideas. In addition, we made a conscious effort to lay aside our personal prior ideas of what to expect. Thus, we consider the findings to have adequate internal validity (Malterud term for trustworthiness)^26^. We recruited women through the internet, mainly Facebook. This may have influenced the sample included, i.e. the youngest not using Facebook much. However, we have a large number of participants from all over the country, thus it is likely that the findings are generalizable to other women in Norway. While some comments were short, women tended to be direct, therefore, even short comments were valuable. It is important to remember that women have answered an elaborate set of questions prior to commenting freely. Thus, the comments are most likely about issues of real importance to women not covered satisfactorily in the quantitative part. Most of the comments requested improvements in the quality of care. Women were free to provide positive and negative comments. Women with negative experiences may be more likely to comment. The number and length of the comments showed that this is an important topic to women.

Our experience with qualitative research and our knowledge of the theory, including previous research on the topic of contraception, is limited. This may have limited the level of abstraction and finding of new concepts. The findings in this study are descriptive.

## CONCLUSIONS

Our study found that the encounter between women and health professionals for contraceptive counselling needs to be improved. The quality can be increased by better communication and sharing of information, between women and care providers, with more questions being asked and tailored information given. Side-effects are important to women and need to be discussed honestly and thoroughly with women and follow-up offered. Non-hormonal methods need to be considered. Sensitivity is required when broaching the topic of sexual health and respect shown, if women do not want to discuss this. All our findings are relevant for clinical practice. However, the most important implication for clinical practice is that in order to achieve a satisfactory consultation enough time needs to be set aside. The competencies of midwives in regard to contraceptive consultations should be made widely known in Norway and thus used more to provide women with continuity of care throughout their reproductive life.
